# Determination of Sorbic Acid in Cheese Samples by Rapid HPLC-DAD Method

**DOI:** 10.1155/2020/6049028

**Published:** 2020-01-23

**Authors:** Ayşe Özdemir, Senem Şanlı, Barış Sardoğan, Seyfi Sardoğan

**Affiliations:** ^1^Division of Biochemistry, School of Medicine, Uşak University, 64100 Uşak, Turkey; ^2^Department of Chemistry, Faculty of Science and Arts, Usak University, Uşak, Turkey; ^3^İzmir Kavram Vocational College of Higher Education, İzmir, Turkey; ^4^Department of Food Engineering, Graduate School of Natural and Applied Sciences, Usak University, Uşak, Turkey

## Abstract

The sorbic acid and its salts have been widely used in the food industries for many years as important food preservatives in order to inhibit the growth of various bacteria, yeasts, and fungi in acidic media. The health effects have led to limitation on the concentrations that can be used in food. Because of that, the analytical determination of these preservatives is important for consumer interest and protection. The purpose of this study is to determine the concentration of sorbic acid in cheese samples by using HPLC. For HPLC analysis, an X-Terra RP-18 (150 × 4.60 mm i.d. × 5 *μ*m) column was selected as the stationary phase at 25°C. Analysis time is about 3 minutes. The developed method was applied to 10 different cheese samples collected from the Turkish market. The levels of sorbic acid in the analyzed samples were between 21.3 mg/kg and 511.3 mg/kg.

## 1. Introduction

Preservatives have been commonly used as additives in food, cosmetics, and pharmaceutical products. Addition of preservatives prevents the alteration and degradation of the product formulation [1]. Nowadays, this type of preservation is often performed with the use of chemical preservatives, such as sorbic acid and its respective sodium, potassium, and calcium salts due to its high solubility. Practical usage of sorbates includes the protection of human food, animal nutrition, pharmaceuticals, cosmetic products, and packaging materials. Sorbates are used as a food preservative in the application areas of cheese and cheese products, yogurt, and sour cream [2–5].

These compounds are generally used to inhibit yeast and mold growth. Additionally, they are effective against a wide range of bacteria. The highest activity of these compounds is recorded in foods with low pH value, while they are noneffective in foods at neutral pH value [6, 7]. Their solubility in water varies depending on the pH and temperature of the environment. As the concentration of soluble food components such as sucrose, glucose, and NaCl increases, the solubility of sorbic acid in water decreases. While the solubility of sorbic acid in water at 25°C is 0.16%, the solubility of potassium sorbate under the same conditions is above 50%. Potassium sorbate with a chemical structure of CH_3_CH = CHCH = CHCOOK is a white crystalline powder. Its solubility in water is very high, and it has a solubility capacity of 139.2 g in 100 ml of water. 20 g dissolves in 1 ml of alcohol at 20°C [8]. Sorbates are more soluble in alcohol compared to water.

Sorbic acid can be differently applied in foodstuffs. It can be added directly to the product or sprayed onto the surface, sprinkled in powder form, dipped into food-grade sorbate solutions prepared in certain concentrations, or coated with sorbate packaging materials. High concentration solutions are required for dipping and spray applications [9].

Food additives such as preservatives may cause an allergic or intolerance reaction. As a preservative, sorbic acid is regarded as safe and nontoxic, but using especially in large amounts can potentially lead to allergies. Migraine, a common type of headache, is one of the possible adverse health effects of potassium sorbate. Higher than normal levels of potassium in the blood may lead to hyperkalemia [10].

The use of sorbic acid and its salts in processed foods is extremely important. Not using this antimicrobial agent may cause microbial activities that lead to food poisoning [11]. However, there are some limitations in using these preservatives. Fermented products are the foremost food group that have limitations for food additives because of their importance in healthy nutrition, prevention, and curing effects. Turkish Food Codex which is prepared considering scientific truths and conclusions of Codex Alimentarius and in accordance with European Union directives is effective in such applications in Turkey [12].

There are various methods for analysis of sorbic acids in food products, such as ultraviolet (UV) spectroscopy, high-performance liquid chromatography (HPLC), GC, and LC-MS/MS [13–22]. The purpose of this study is to determine the concentration of sorbic acid in cheese samples by using HPLC. HPLC detection has become the most widely applied analytical separation technique because of its superior performance and reliability, especially in the pharmaceutical, environmental, forensic, clinical, food, and flavor sciences.

## 2. Experimental

### 2.1. Chemicals

All chemicals in this study were used without further purification. The standard of sorbic acid was obtained from Sigma-Aldrich (St. Louis, Missouri, USA). HPLC-grade acetonitrile (ACN) and methanol (MeOH) were purchased from Merck. Ultrapure water, with conductivity lower than 0.05 *μ*S/cm, was obtained with a Milli-Q system (Millipore, Bedford, MA, USA).

Stock solutions of sorbic acid were prepared by dissolving in water (50 mL) to make a 100 mg/L solution. All solutions were protected from light, and all solutions were stored at 4°C for short time usage (daily) and at −20°C for long-term usage (between days).

### 2.2. Apparatus

The HPLC analysis was carried out on an Agilent 1260 series HPLC system with ternary solvent pump, online degasser, automatic injection system, column heater, and multi wavelength detector. UV detection was performed at 250 nm. Analyses were run at a flow rate of 1.0 mL·min^−1^. Because of the peak shape and analysis time, an X-Terra RP-18 (150 × 4.60 mm i.d. × 5 *μ*m) column was selected as stationary phase at 25°C. 50% (*v*/*v*) ACN-water containing 0.2 (*v*/*v*) glacial acetic acid was used as a mobile phase.

### 2.3. Preparation of Cheese Samples for HPLC Analysis

Ten commercial cheese samples were purchased from local Turkish markets in Uşak. A cheese sample (5.00 ± 0.01 g) was weighed in a conical flask and 20 ml of water was added to this sample. The mixture was stirred for 20 minutes in stomacher and then 35.0 ml of methanol was added to this solution. The volume was made up to 100 ml with deionized water. A portion of the filtrate was filtered through a membrane microfilter of pore size 0.45 *μ*m (Minisart RC25 17765) and then 0.20 *μ*m (Minisart RC25). The resulting filtrate was used for 20 *μ*L per injection for chromatographic analysis. The peak area was obtained for each solution on the ordinate against the sorbic acid concentration, in milligrams per liter, on the abscissa.

## 3. Results and Discussion

The use of sorbic acid and its salts in processed foods is extremely important. Not using this antimicrobial agent may cause microbial activities that lead to food poisoning [11]. However, there are some limitations. Fermented products are the foremost food group that have limitations for food additives because of their importance in healthy nutrition, prevention, and curing effects. Turkish Food Codex that is prepared considering scientific truths and conclusions of Codex Alimentarius and in accordance with European Union directives is effective in such applications in Turkey [12].

**Figure 1:**
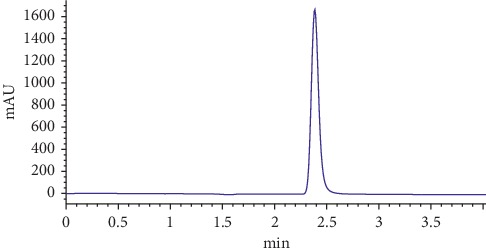
Chromatogram of a 50 *μ*g·mL^−1^ sorbic acid standard. The eluent was monitored at 250 nm. Mobile phase consists of ACN : water (50 : 50, *v*/*v*) containing 0.2 (*v*/*v*) acetic acid. Flow rate is 1 mL·min^−1^.

This paper presents rapid and simple methods for the determination of sorbic acid in 10 cheese samples by UV-DAD. The improved HPLC method has a good resolution with a short analysis time. For the determination of sorbic acid in cheese samples, C18 column of (150 mm × 4.6 mm) dimension and 5 *μ*m of particle size was used. The mobile phase used during the analysis was 50% (*v*/*v*) ACN-water containing 0.2 (*v*/*v*) glacial acetic acid. By this chromatographic conditions, analysis time is about three minutes (Figure 1).

The chromatographic process was performed at 250 nm. The requirements for the system suitability are usually designed after the development and validation of the method have been completed. The system suitability test results are given in Table 1.

A set of sorbic acid standards were tested to determine the validation parameters (linearity, range, detection limit, and quantitation limit) [23]. The linearity was calculated by plotting the peak area versus concentration of sorbic acid. Five solutions were prepared with 1.00 *μ*g·mL^−1^, 5.00 *μ*g·mL^−1^, 10.0 *μ*g·mL^−1^, 20.0 *μ*g·mL^−1^, and 50.00 *μ*g·mL^−1^ sorbic acid concentration, respectively. Each solution was injected in duplicate. The calibration curves were obtained by linear least-squares regression. The validation data are reported in Table 2. The method exhibited good linearity based on a correlation coefficient >0.999 for sorbic acid. The LOD and LOQ were calculated as 3.3 s·m^−1^ and 10 s·m^−1^, respectively, where *s* is the standard deviation of the response and *m* is the slope of the corresponding calibration curve [24, 25].

Repetitive analyses of standard solutions containing 5–20 *μ*g mL^−1^ sorbic acid were performed to evaluate the precision and reproducibility of the method. Sorbic acid was analyzed in consecutive days with five replicates. Repeatability and reproducibility were characterized by mean recovery and RSD, and the results are summarized in Table 3. As deduced from Table 3, there was no significant difference for the assay, as tested by within-day (intraday) and between-days (interday) precision. The results supported good precision of the method.

**Figure 2:**
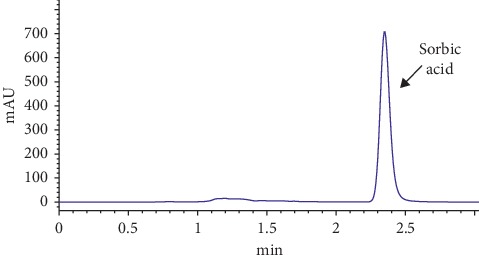
Chromatogram of kashar cheese samples.

The method was finally tested to evaluate the content of sorbic acid in several kinds of commercial cheeses, and the data are shown in Table 4. As shown in Table 4, in all of the analyzed fresh and kashar cheese samples, the detectable level of sorbic acid was determined. Sorbic acid levels in the cheese samples were found to be lower than the maximum acceptable limits (matured cheese: 1000 mg·kg^−1^ or L^−1^) of the Turkish Food Codex [26]. In Figure 2 , kashar cheese chromatogram is given as an example.

For the accuracy defined by developed method, known amount of sorbic acid was spiked to the cheese sample and analyzed by the developed method. The study was done at 5 mg/kg and 10 mg/kg of test concentration levels. Recovery % was determined on *n* = 10 analyzed samples for each spiking level. The results were found to be 97.5 and 98.1 with low level of RSD values at 0.314 and 0.210, respectively. Sufficient precision and accuracy could be achieved by this developed method for analysis of cheese samples. The high recovery values obtained show that the method is not affected by the matrix effects in cheese samples.

In the literature, the HPLC method was used for the determination of benzoic acid, sorbic acid, methylparaben, and propylparaben in foodstuffs by Saad et al. [13]. Analysis time for the separation is about 20 minutes, and LOD and LOQ values are 0.1 and 0.3 mgL^−1^, respectively. Petanovska-Ilievska et al. [14] reported an LOD of 0.003 mgL^−1^ with the analysis time of 5 minutes. The HPLC method of Zor et al. [15] reported that LOD and LOQ for sorbic acid were 0.082 and 0.271 *μ*g·mL^−1^, respectively. The method described above provides a combination of faster analysis time and improved limits of detection.

## 4. Conclusions

Sorbic acid is widely used in milk products in Turkey. Therefore, the use of sorbic acid by dairy companies should be supervised by the Turkish public health authorities.

In this study, a novel and fast HPLC method for the determination of sorbic acid in 10 commercial cheese samples was developed with good accuracy and precision. The analysis is completed in about 3 minutes.

High recovery values indicate that the method is independent of the detrimental effects of additives commonly used in cheese samples. This study has lower detection limit and higher recovery values compared with literature data.

## Figures and Tables

**Table 1:** System suitability parameters of the proposed HPLC method.

Parameters	DAD
Retention time (min)	2.45 ± 0.19
Theoretical plates (N)	3758 ± 145
Tailing factor	0.97 ± 0.01
Peak wavelength	0.11 ± 0.02

**Table 2:** Statistical evaluation of the calibration data of sorbic acid by HPLC-DAD method.

	Sorbic acid
Linearity range (*μ*g·mL^−1^)	1.00–50.00 (*n* = 5)
Slope	173.79
Intercept	36.592
Correlation coefficient (*r*)	0.999
Limit of detection (LOD) (*μ*g·mL^−1^)	0.0018
Limit of quantitation (LOQ) (*μ*g·mL^−1^)	0.0269

**Table 3:** Summary of repeatability (intraday) and reproducibility (interday) precision data for sorbic acid by HPLC-DAD.

Compound concentration (*μ*g·mL^−1^)	Intraday	Interday
	Mean recovery^*∗*^ % ± RSD %	Mean recovery^*∗*^ % ± RSD %
5.0	100.115 ± 0.851	100.025 ± 0.651
20.0	100.240 ± 0.112	99.918 ± 0.498

^*∗*^Each value is obtained from five experiments (*n* = 5).

**Table 4:** Sorbic acid content in cheese samples collected from local markets.

Sample	
Kashar cheese 1	244.90 mg/kg
Kashar cheese 2	271.5 mg/kg
Kashar cheese 3	229.05 mg/kg
Kashar cheese 4	511.3 mg/kg
Fresh cheese 5	255.65 mg/kg
Fresh cheese 6	100.10 mg/kg
Fresh cheese 7	99.80 mg/kg
Fresh cheese 8	72.10 mg/kg
Fresh cheese 9	21.30 mg/kg
Fresh cheese 10	28.40 mg/kg

## Data Availability

The data used to support the findings of this study are included within the article.
